# Book Reviews

**Published:** 1905-02

**Authors:** 


### BOOK REVIEWS.

Hare's Practice of Medicine. A text-book for students and practitioners. By Hobart Amory Hare, M.D., B.Sc.; Professor of Therapeutics and Materia Medica in the Jefferson Medical College of Philadelphia; Physician to the Jefferson Medical College Hospital; Laureate of the Royal Academy of Medicine in Belgium; of the Medical Society of London; author of A Text-Book of Practical Therapeutics, A Text-Book of Practical Diagnosis, etc. In one very handsome volume of about 1,000 pages, with about 100 engravings and 6 full-page plates in colors and monochrome.

As the student of to-day is the physician of the future, and as the physician must always be a student, a single volume can be conceived as answering the requirements both of a text-book and work of reference. To produce such a volume the author has brought to bear his experience of twenty years of active hospital and private practice, during which period he has been constantly engaged in teaching the subjects of clinical medicine and therapeutics. This didactic work has enabled him to understand the difficulties which confront the student, and the necessity of presenting the principles and data with the utmost clearness. If the author has succeeded in impressing this quality upon his pages they will be none the less serviceable to graduate readers.

The book has purposely been given a clinical character. For this reason illustrations and plates have been introduced wherever an important point could be made more clear than by verbal description.

Pneumonia and Pneumocoecus Infections. By Robt. B. Preble, A.B., M.D., Prof. of Medicine, Northwestern University, Chicago. Cloyd J. Head & Co., 40 Dearborn St. 1905.

In this volume of 211 pages the author has presented very fully the modern acceptation of this disease, and has given also the benefit of his wide experience as a clinicil and teacher. The fact that pneumonia is in the first rank in the point of mortality renders it imperative that every physician should endeavor to secure a thorough knowledge of it and put himself in possession of all known facts concerning it, in order to enable him to cope with it intelligently. His book offers this opportunity.

---

THE SUPPRESSION OF TUBERCULOSIS. Together with Observations Concerning Phthisiogenesis in Man and Animals, and Suggestions Concerning the Hygiene of Cow Stables and the Production of Milk for Infant Feeding, with Special Reference to Tuberculosis. By Prof. E. von Behring (University of Marbury). Authorized Translation, by Charles Baldwin, M.D. New York, John Wiley & Sons.

This volume consists of a translation of lectures and articles by Prof. von Behring, giving his views on the genesis and suppression of tuberculosis. This is an all-important question, and the opinion of such a distinguished authority as von Behring will be received and utilized by the profession as primal aids in the warfare that is being actively waged against this greatest of plagues.

---

NEW METHODS OF TREATMENT. By Dr. Laumonier. Translated and Edited from the Second Revised and Enlarged French Edition by H. W. Syers, M.A., M. D. Canton. Chicago, W. T. Keener & Co., 90 Wabash Ave. 1904.

The object of this book is to collect from current literature new remedies, new theories and new methods in the treatment of disease. Naturally there is much that is ephemeral, but judgment and experience have been elicited in the selection of the material that was deemed worthy of consideration. The book will prove of great benefit to him who is desirous of acquainting himself with present day materia medica and therapeutics without having recourse to over-discursive medical journal reading.

